# Congenital scrub typhus: a case report and literature review

**DOI:** 10.3389/fped.2023.1251746

**Published:** 2023-11-20

**Authors:** Pinghua Liang, Zengling Su, Min Chen, Sitao Li

**Affiliations:** ^1^ Department of Pediatrics, Yuexi Hospital of the Sixth Affiliated Hospital, Sun Yat-sen University (Xinyi People’s Hospital), Xinyi, China; ^2^ Department of Pediatrics, the Sixth Affiliated Hospital, Sun Yat-sen University, Guangzhou, China; ^3^ Biomedical Innovation Center, the Sixth Affiliated Hospital, Sun Yat-sen University, Guangzhou, China

**Keywords:** congenital scrub typhus, vertical transmission, clinical manifestations, early diagnosis, early treatment

## Abstract

**Background:**

This study aimed to analyze the clinical course of a newborn with congenital scrub typhus caused by vertical transmission and explore early diagnosis and treatment strategies. The clinical data of the neonate were retrospectively analyzed and the related literature was reviewed.

**Case presentation:**

The newborn was a full-term one, with a good Apgar score at birth. The neonate had apnea at 3 h of life, requiring NICU care and IV antibiotics (piperacillin) for suspected sepsis. An examination revealed hepatosplenomegaly. Blood tests revealed anemia and thrombocytopenia and a chest x-ray showed patchy inflammation. On the second day of life, he developed a fever. On the third day of life, he required mechanical ventilation because his condition worsened after he presented with dyspnea, hypotension, depressed sensorium, and other signs of sepsis. Importantly, the neonate's mother had a history of scrub typhus at 31^+^ weeks of gestation. While the blood culture result was still pending, high-throughput sequencing of blood and cerebrospinal fluid was performed. To address the suspected scrub typhus infection, oral azithromycin dry suspension was added to the treatment regimen. High-throughput sequencing results on the 5th day of life confirmed a significant presence of 16SrRNA sequences in the blood, suggesting an *Orientia tsutsugamushi* infection. The neonate steadily recovered and was discharged 16 days after hospitalization. The neonate was followed up for 9 months, and the outcome was favorable with normal growth and development.

**Conclusions:**

This article reports a case of congenital *Orientia tsutsugamushi* infection, a rare condition caused by vertical transmission. Our review of the literature, combined with the presented case, brings the total number of documented congenital scrub typhus cases caused by vertical transmission to eight. Regrettably, one patient from this group unexpectedly died on the 10th day of hospitalization, resulting in a mortality rate of 12.5% (1/8). The special transmission mode and clinical manifestations of this disease will serve as an alert to doctors for timely diagnosis and treatment. Because of the non-specific clinical manifestations of congenital scrub typhus, limited understanding, low index of suspicion among clinicians, and a lack of diagnostic facilities, scrub typhus is seriously underdiagnosed in pregnant women, fetuses, and neonates.

## 1. Background

Scrub typhus, also known as tsutsugamushi disease, is a zoonotic disease caused by the gram-negative obligate intracellular pathogen *Orientia tsutsugamushi* (formerly known as Rickettsia tsutsugamushi until 1995), which is mainly present in tropical and subtropical regions and is transmitted by mites (or chiggers) (1). Scrub typhus can manifest with various symptoms such as fever, headache, muscle pain, rash, and organ failure. Scrub typhus is one of the most common recurrent vector-borne diseases (2). While humans are incidental hosts, nearly 1 million children are infected annually (3). However, cases of vertical transmission are rare. Timely diagnosis is crucial due to the occurrence of non-specific early clinical symptoms because delayed recognition can lead to severe complications such as acute respiratory distress syndrome (ARDS), septic shock, multiple organ failure, and even death. There are only a few clinical reports and studies on congenital scrub typhus in China and worldwide. Only eight cases of congenital scrub typhus have been reported (4–10), leaving aspects of its clinical presentation and treatment relatively unexplored. In this study, we discuss the clinical course of a newborn with congenital scrub typhus and review relevant literature to gain insights into the clinical characteristics and treatment of this condition.

## 2. Case presentation

The male infant, G1P1, with a gestational age of 39^+1^ weeks, was delivered by cesarean section because of fetal distress, at Xinyi People's Hospital of Guangdong Province on 12 August 2022. The mother had an uneventful prenatal history, with no reports of fever, premature rupture of membranes, and gestational diabetes mellitus, and tested negative for group B streptococcus. A placenta and umbilical cord examination showed no abnormalities, and the newborn weighed 2.6 kg. The immediate postnatal assessment indicated a full-term baby with meconium-stained amniotic fluid, heart rate >100 beats/min, good muscle tone, and vitality, and he exhibited loud crying. Routine newborn care such as providing warmth, cleaning the respiratory tract, drying the whole body, and stimulation was administered. The Apgar score was 10 at 1 min, 10 at 5 min, and 10 at 10 min. The neonate had apnea at 3 h of life, requiring neonatal intensive care unit (NICU) care and IV antibiotics (piperacillin) for suspected sepsis. An examination revealed hepatosplenomegaly. Blood tests revealed anemia and thrombocytopenia and a chest x-ray showed patchy inflammation in both lungs. Blood gas test results showed a PH of 7.23, PO_2_ of 55 mmHg, a PCO_2_ of 60 mmHg, a base excess (BE) of −6.6, and a lactic acid level of 7 mmol/L. In addition to sepsis, other viral infections were considered, with samples being taken for herpes simplex virus, cytomegalovirus, rubella virus, *Toxoplasma gondii*, and COVID-19; all of the test results proved negative. On the second day of life, the infant developed a fever of 38.5°C. Blood tests showed an increased C-reactive protein (CRP) level of 113.24 mg/L, white blood cell count (WBC) was normal, hemoglobin (Hb) level was 117.0 g/L, and platelet count (PLT) was 58 × 10^9^/L. Antibiotic treatment was changed to meropenem (discontinued on the eight day of life) and vancomycin (discontinued on the sixth day of life). On the third day of life, his condition worsened and he presented with dyspnea, hypotension (42/21 mmHg), depressed sensorium, and other signs of sepsis, following which he required mechanical ventilation. A cardiac ultrasound revealed mild persistent pulmonary hypertension (tricuspid regurgitation peak gradient was 39 mmHg). Further history-taking revealed that the child's mother had been diagnosed with scrub typhus and hospitalized at 31^+^ weeks of gestation. She had an eschar on the left neck, approximately 3 mm × 3 mm in size, surrounded by a red halo, and received treatment with intravenous azithromycin for 3 days, followed by 4 days of oral azithromycin after the subsistence of fever. Unfortunately, she did not adhere to the prescribed treatment regimen after discharge, stopping medication just one day later. Because of this complex maternal history, a blood and CSF sample was taken for high-throughput sequencing (also known as next-generation sequencing, NGS), and oral azithromycin dry suspension was administered before the results became available. Blood tests on day 4 showed an elevated CRP level of 161 mg/L, Hb of 108.0 g/L, and PLT of 18 × 10^9^/L. Apheresis platelets were used to improve the platelet count, and methylprednisolone was administered to reduce inflammation. On day 5, blood tests revealed a CRP of 164 mg/L, Hb of 103.0 g/L, and PLT of 43 × 10^9^/L. High-throughput sequencing confirmed a significant presence of 16SrRNA sequences in the blood, confirming an *Orientia tsutsugamushi* infection. The infant continued to receive oral azithromycin treatment, and the results of subsequent blood culture tests were negative. He showed clinical improvement on the sixth day of life, as evidenced by sensorium improvement, normal blood pressure level, and spontaneous breathing. After this, the neonate's condition gradually improved, CRP gradually decreased, and platelets gradually recovered, following which he was weaned from ventilator inhalation to be placed on nasal catheter oxygen inhalation on the seventh day of life; on the 10th day of life, oxygen inhalation was also stopped. The neonate recovered and was discharged on the 16th day of life (Table 1). He was followed up for 9 months, and the outcome was favorable, with normal growth and development.

**Table 1:** Clinical course and crucial changes in routine blood test results.

Date	Clinical symptom	Respiratory/vasopressor support	Mode of feeding/nutrition	Sensorium	Hb (g/L)	PLT × 10^9^/L	CRP (mg/L)
8–12 3 h after birth	Cyanosis, slow heart rate	Nasal oxygen	Nasal oxygen	Normal	126	89	—
2nd day	Fever, 38.5°C	Nasal oxygen	Nasal oxygen	Normal	117	58	113.24
3rd day	Dyspnea, sepsis	Tracheal intubation, ventilator-assisted ventilation	Fasting, intravenous nutrition	Depressed	86	52	>200
4th day	Abnormal coagulation function, diffuse intravascular coagulation	Ventilator-assisted ventilation, dopamine	Fasting, intravenous nutrition	Depressed	108	18	161.41
6th day		Ventilator-assisted ventilation, dopamine	Gastric tube input milk, partial intravenous nutrition	Improved	160	26	69.62
7th day		Ventilator-assisted ventilation	Gastric tube input milk, partial intravenous nutrition	Improved	161	58	35.34
8th day		Nasal oxygen	Nasal oxygen	Normal	155	78	19.85
11th day		Stopping oxygen	Nasal oxygen	Normal	133	128	12.32
16th day	Hospital discharge				125	235	7.35

## 3. Discussion and conclusions

The search terms “tsutsugamushi disease,” “neonatal tsutsugamushi disease,” “vertically transmitted tsutsugamushi disease,” “congenital tsutsugamushi disease,” “scrub,” “typhus,” “vertically transmitted scrub typhus,” “congenital scrub typhus,” “rickettsia tsutsugamushi,” and “*Orientia tsutsugamushi*” were employed when conducting searches on PubMed and China National Knowledge Infrastructure (CNKI) from the period of inception of the databases up to August 2023. We collected and analyzed clinical and follow-up data for children with complete information. The comprehensive literature review, in conjunction with the present case, yields a total of eight cases of congenital scrub typhus resulting from vertical transmission (as detailed in Table 2) (4–10). In their study, Wang et al. (4) reported the case of a premature infant born at 34 weeks of gestation, while the remaining cases involved full-term infants. The common clinical features observed in almost all infants were fever, hepatosplenomegaly, and abnormal liver function. Anemia was reported in three infants, sepsis in two, thrombocytopenia in two, and disseminated intravascular coagulation in three others. Treatment approaches varied, with azithromycin, doxycycline, and chloramphenicol being administered. In two infants, doxycycline was given initially following diagnosis, and azithromycin was added when the initial treatment proved ineffective. Unfortunately, one patient from this group unexpectedly died on the 10th day of hospitalization. Considering the outcome of these eight cases, we can extrapolate that the mortality rate of congenital scrub typhus could be approximately 12.5% (1/8). However, four of them had a favorable outcome without any long-term complications. Detailed follow-up data were not available for the remaining patients.

**Table 2:** Demographic and clinical characteristics of 8 neonates with congenital scrub typhus.

Cases	Age of onset	Sex	Symptom	Physical examination	Maternal symptoms	Lab confirmation	Outcome	Treatment
Our case	3 h	Male	Acute respiratory distress, fever, anemia, sepsis, thrombocytopenia, and disseminated intravascular coagulation, without petechiae or eschar	Hepatosplenomegaly, thrombocytopenia with the lowest value of 18 × 10^9^/L; abnormal coagulation function; ALT, 71.7 IU/L; AST, 132.0 IU/L; cerebrospinal fluid: WBC 96 × 10^6^/L; RBC 1,000 × 10^6^/L; monocytes, 96%; protein, 4.12 g/L; glucose, 2.38 mmol/L	Fever and left neck eschar at 31^+^ weeks of gestation	Fluorescence immunoassays were not performed. High-throughput sequencing of peripheral blood showed that the pathogen was *Orientia tsutsugamushi*	Recovery without sequelae	Azithromycin
Wang et al. (4)	26 days	Male	Intermittent high fever, abdominal distention, and pale skin without petechiae or eschar	Hepatosplenomegaly; AST, 92 IU/L; ALT, 54 IU/L; cerebrospinal fluid, 350 cells/high-power field (170 red cells, 170 white cells, 180 polymorphisms, 63% mononuclear cells); protein, 128 mg/dl; glucose, 79 mg/dl	Fever on postnatal day 4 that lasted for 1 week	Infant: The Weil–Felix test OXK 1:320, IgM-ELISA 1:160 (Kato), Mother: elevated specific IgM-ELISA antibodies against Karp and Gilliam	Complications of meningitis and disseminated intravascular coagulopathy	Doxycycline
Suntharasaj et al. (5)	4 days	Male	Anemia, sepsis, and disseminated intravascular coagulation were present, with no petechiae or eschar	Hepatosplenomegaly	High fever, chills, dry cough, headache, and pneumonia	Infant: The Weil–Felix test OXK 1:320, IgM-ELISA 1:400 (Kato). Mother: The Weil–Felix test OXK 1:320, IgM-ELISA 1:400 (Kato)	Mild growth retardation and encephalomalacia occurred after recovery	Chloramphenical
Vajpayee et al. (6)	23 days	Male	Jaundice, abdominal distention, low-grade fever, and no petechiae or eschar were present	Hepatomegaly was accompanied by acute liver failure	Not described	Infants and mothers: elevated specific IgM-ELISA antibodies	Recovered without sequelae	Azithromycin
Gao et al. (7)	4 days	Male	Intermittent high fever, jaundice, and convulsions occurred without petechiae or eschar	Hepatomegaly; AST, 336.6 U/L; ALT, 1,317.1 U/L; cerebrospinal fluid: white blood cells, 283 × 106 /L; monocytes, 95.1%; neutrophils, 4.9%; protein, 3.08 g/L; glucose, 1.18 mmol/L	Anemia and reduced fever with eschar	Infant: The Weil–Felix test OXK 1:160, IgM-ELISA 1:160 (Kato). Mother: The Weil–Felix test OXK 1:640, IgM-ELISA 1:160 (Kato).	Recovered without sequelae	Azithromycin
Deglurkar et al. (8)	36 h	Female	Fever, irritability, hepatosplenomegaly, tachycardia, tachypnea, prolonged capillary refill time, and meningitis.	Thrombocytopenia, direct hyperbilirubinemia, and transaminitis	Mother had developed a febrile illness with a rash at approximately 34 weeks of gestation. She was treated with 5 days of oral azithromycin.	Infant: positive scrub typhus PCR. Mother: positive scrub typhus PCR	Recovered without sequelae	Doxycycline and azithromycin
Ganesh et al. (9)	Not provided	Not provided	Not provided	Not provided	Not provided	Scrub typhus IgM-ELISA was positive scrub typhus PCR	Not provided	Doxycycline
Aarthi et al. (10)	At birth	Female	Respiratory distress, fever, lethargy, seizures, squint, petechiae, and hepatosplenomegaly	Platelet count decreased, Liver enzymes Elevated	Presented with fever with hypotension for 5 days in the 7th month of pregnancy, Reduced fetal movement in the last week of pregnancy, delivered at term, Meconium-stained liquor	Positive scrub typhus PCR	died on the 10th day of hospitalization	Doxycycline and azithromycin

Scrub typhus is the most prevalent human rickettsial infection caused by *Orientia tsutsugamushi* which is transmitted through the bites of infected mites. In the wild, *Orientia tsutsugamushi* survives inside mites (the main vector) and other vertebrates (small mammals and birds), and humans are incidental hosts (11). This infection is commonly seen in people exposed to the vector as a result of their occupational activities and in people living in the vicinity of a wide range of vegetation types, ranging from scrubs (terrain between woods and clearing) and primary forests to gardens and beaches (12). China is one of the primary endemic regions for scrub typhus, especially in the Yunnan and Guangdong provinces (13, 14). The annual incidence rates in these provinces among children aged 0–9 years were 2.46/100,000 and 4.8/100,000, respectively. The regions with the highest incidence rates were Baoshan, Dehong, Lincang, and Xishuangbanna in southwestern Yunnan Province, with the rates being as high as 52.48/100,000 (15).

Scrub typhus is rarely reported during the neonatal period. The modes of transmission in neonates may include mite-borne transmission, trans-placental transmission, and perinatal blood-borne transmission (16). A brief literature review reveals 22 reported cases of neonatal scrub typhus (8). Most cases were attributed to mite-borne transmission, while a few were associated with trans-placental transmission. To date, there has been no reported case of blood-borne transmission. The pathogen enters the body and primarily targets the endothelial and reticuloendothelial cells, leading to diffuse and perivascular vasculitis. Vasculitis is the basic pathogenesis that leads to symptoms such as rash, microvascular leakage, edema, tissue hypoperfusion, and end-organ ischemic damage (16). Extensive tissue hypoxic–ischemic injury can lead to multiple organ dysfunction, resulting in high morbidity and mortality in untreated cases (17, 18). The incubation period of *Orientia tsutsugamushi* in humans typically ranges from 10 to 21 days. In this review, fever, hepatosplenomegaly, and abnormal liver function were observed in almost all patient cases. However, of the other neonates with congenital disease, a few had altered liver function and organomegaly–hepatomegaly. The clinical manifestations can range from mild undifferentiated fever to severe and potentially fatal disease, which can progress to multiple organ dysfunction syndrome (MODS).

The laboratory diagnosis of scrub typhus involves several assessment methods, including antibody detection by an indirect immunofluorescence test, indirect immunoperoxidase test, enzyme-linked immunosorbent assay (ELISA), and immunochromatography test (ICT), genome detection by polymerase chain reaction (PCR), and next-generation sequencing (NGS). Among these diagnostic methods, molecular-based methods such as PCR and NGS exhibit higher specificity and sensitivity (19). However, during the acute stage of neonatal scrub typhus, the results of the serological test often turn out to be negative, which can lead to misdiagnosis and a high risk of complications and mortality (8). Therefore, it is crucial to consider scrub typhus as a potential diagnosis in children and initiate prompt treatment (20).

A summary of the eight congenital cases presented in this study unveiled some characteristics as well as some doubts. First, most patients had only non-specific symptoms, with none presenting eschar or rash. Delivery occurring in an endemic area may be the only clue for tracing exposure history. Therefore, a detailed investigation of the medical history of pregnant women is required. Second, scrub typhus infection in pregnant women is associated with poor fetal outcomes; maternal infection in the first and second trimesters, a higher risk of fetal loss, and premature delivery (21). Therefore, prevention and intervention of scrub typhus during pregnancy can reduce the risk of adverse fetal and neonatal outcomes. Third, elevated IgM during the first weeks of life provides direct evidence of intrauterine infection. *Orientia tsutsugamushi* invades the vascular endothelium, leading to plasma leakage and end-organ ischemia. However, the pathogenic mechanisms associated with placental vasculitis remain unclear, and they may be associated with thrombotic occlusion and coagulopathy (22). Finally, all patients with congenital scrub typhus in this study had severe clinical symptoms and/or complications, which may be attributed to the origin of congenital scrub typhus from prolonged intrauterine infection and/or delayed diagnosis.

Scrub typhus is a treatable condition with antibiotics, but if left untreated, it can lead to MODS and even death. Hence, early diagnosis is imperative. The antibiotics commonly used to treat scrub typhus are tetracycline, macrolides, chloramphenicol, fluoroquinolones, and rifampicin. However, experts have varying opinions on which drugs to recommend for newborns. The American Academy of Pediatrics Committee on Infectious Diseases revised its recommendations in 1997 and identified doxycycline as the drug of choice for treating presumed or confirmed Rocky Mountain spotted fever (RMSF) in children of any age (23). Doxycycline is the drug of choice in a dose of 100 mg twice daily (BID) in children above 45 kg and 4 mg/kg/day in two divided doses for children below 45 kg (23). Treatment should continue until the patient becomes afebrile for 3 days or 7–10 days. However, some experts disagree with doxycycline as the first choice of treatment, particularly in neonates, because of its potential side effects, and instead prefer azithromycin and clarithromycin (24). Macrolides, including azithromycin, are considered safe for use during pregnancy and in young children, with a recommended dose of 10 mg/kg/day (maximum 500 mg) for 5 days (2, 25, 26). Furthermore, azithromycin seems to be a favorable option in pregnant women with scrub typhus (27). In our case, the mother received inadequate azithromycin treatment (3 days intravenous plus 1 day oral), which might have contributed to the vertical transmission. The treatment of scrub typhus is straightforward and effective when diagnosed early. The mortality rates can be as high as 30% in untreated or inadequately treated cases (16). Even with treatment, death may occur depending on the severity of complications (16). Based on this case and the previous literature review, it is crucial to emphasize that neonates presenting with fever, respiratory distress, hepatosplenomegaly, edema, thrombocytopenia, and shock without signs of septicemia or congenital infection should undergo investigation for scrub typhus when epidemiological risk is present. When clinically suspected, diagnostic treatment can be initiated empirically, especially in regions where scrub typhus is endemic.

Currently, there are only a few reports of congenital scrub typhus. The special transmission mode and clinical manifestations of this disease serve as an alert to doctors for timely diagnosis and treatment. Because of the non-specific clinical manifestations of congenital scrub typhus, limited understanding, low index of suspicion among clinicians, and a lack of diagnostic facilities, scrub typhus is seriously underdiagnosed in pregnant women, fetuses, and neonates. Further studies are required to shed more light on this disease, particularly in endemic areas.

## Data Availability

The original contributions presented in the study are included in the article/Supplementary Material, further inquiries can be directed to the corresponding authors.
